# Genome sequencing and comparative genome analysis of *Rhizoctonia solani* AG-3

**DOI:** 10.3389/fmicb.2024.1360524

**Published:** 2024-04-04

**Authors:** Shanshan Xu, Chengmeng Shen, Chengyun Li, Wenhan Dong, Genhua Yang

**Affiliations:** State Key Laboratory for Protection and Utilization of Bio-Resources in Yunnan, Yunnan Agricultural University, Kunming, Yunnan, China

**Keywords:** *Rhizoctonia solani* AG-3, subgroup, *de novo* sequencing, phylogenetic evolution, pathogenicity-related genes

## Abstract

*Rhizoctonia solani* AG-3 is a plant pathogenic fungus that belongs to the group of multinucleate *Rhizoctonia.* According to its internal transcribed spacer (ITS) cluster analysis and host range, it is divided into TB, PT, and TM subgroups. AG-3 TB mainly causes tobacco target spots, AG-3 PT mainly causes potato black scurf, and AG-3 TM mainly causes tomato leaf blight. In our previous study, we found that all 36 tobacco target spot strains isolated from Yunnan (Southwest China) were classified into AG-3 TB subgroup, while only two of the six tobacco target spot strains isolated from Liaoning (Northeast China) were classified into AG-3 TB subgroup, and the remaining four strains were classified into AG-3 TM subgroup, which had a unique taxonomic status, and there was no previous report on the whole genome information of AG-3 TM subgroup. In this study, the whole genomes of *R. solani* AG-3 strains 3T-1 (AG-3 TM isolated from Liaoning) and MJ-102 (AG-3 TB isolated from Yunnan) isolated from tobacco target spot in Liaoning and Yunnan were sequenced by IIumina and PacBio sequencing platforms. Comparative genomic analysis was performed with the previously reported AG-3 PT strain Rhs1AP, revealing their differences in genomes and virulence factors. The results indicated that the genome size of 3T-1 was 42,103,597 bp with 11,290 coding genes and 49.74% GC content, and the genome size of MJ-102 was 41,908,281 bp with 10,592 coding genes and 48.91% GC content. Through comparative genomic analysis with the previously reported strain Rhs1AP (AG-3 PT), it was found that the GC content between the genomes was similar, but the strains 3T-1 and MJ-102 contained more repetitive sequences. Similarly, there are similarities between their virulence factors, but there are also some differences. In addition, the results of collinearity analysis showed that 3T-1 and MJ-102 had lower similarity and longer evolutionary distance with Rhs1AP, but the genetic relationship between 3T-1 and MJ-102 was closer. This study can lay a foundation for studying the molecular pathogenesis and virulence factors of *R. solani* AG-3, and revealing its genomic composition will also help to develop more effective disease control strategies.

## 1 Introduction

*Rhizoctonia solani* is the most complex and largest multinucleate *Rhizoctonia* in the genus *Rhizoctonia* spp., which has a global distribution and can infect hundreds of plant species. Currently, *R. solani* is classified into 13 fusion groups (AG-1 to AG-13) based on mycelial fusion reaction, and 28 subgroups under the fusion groups based on host, pathogenicity, and ITS sequences (Carling et al., 2002). Among them, the AG-3 fusion group is divided into three subgroups, AG-3 PT (potato type), AG-3 TB (tobacco type) and AG-3 TM (tomato type), according to its host range and ITS sequences, and AG-3 PT can infect all parts of potato, and potato black scurf caused by AG-3 PT has become an important disease affecting the yield and commercial value of potato tubers in the main production areas of potato in various countries (Tsror, 2010). AG-3 TB mainly affects the leaves of tobacco, and it can cause target spot disease of tobacco, which poses a serious threat to the tobacco industry (Wu et al., 2006). AG-3 TM was first reported only in 2020 (Misawa et al., 2020) and mainly causes leaf blight in tomatoes, affecting its growth and development. Among them, tobacco target spot occurs in all major tobacco-producing areas in China. Under natural conditions, the pathogen can produce sexual spores and sclerotia for reproduction and overwintering and can not only parasitize and reproduce on tobacco, but also on rice, eggplant, and pepper-parasitic reproduction (Liu et al., 2020).

The study of its molecular pathogenic mechanism can not only provide potential drug design targets for its prevention and treatment but also provide a reference basis for its molecular genetic breeding. Currently, only studies on the genetic diversity (Ceresini et al., 2002), cell wall degrading enzyme activity (Tuo et al., 2015), and crude extract activity and composition (Kankam et al., 2018) of the AG-3 fusion group of *R. solani* have been carried out, and the pathogenesis is not clear, and the study of its genome is the first step to understand its pathogenic mechanism. A total of 13 *R. solani* from seven fusion groups (AG1 (IA, IB, IC), AG2, AG3 (TB, PT), AG4, AG5, AG6, AG8) have been sequenced. The genome size was ~40 Mbp, and the GC content was about 45%. The differences in secretory proteome and carbohydrate enzyme content between them were also analyzed (Kaushik et al., 2022). However, except for several effector proteins screened in the AG-1 fusion group, the other several have not been studied in depth (Wei et al., 2020).

Previous laboratory studies showed that Yunnan tobacco isolates were categorized into the TB subgroup, while some tobacco isolates from the Liaoning region were categorized into the TM subgroup (Xu et al., 2023a), which has a unique taxonomic status. In this study, we sequenced the genomes of 3T-1 and MJ-102 isolated from tobacco target spot disease in Liaoning and Yunnan using PacBio and Illumina high-throughput sequencing technologies. The phylogenetic tree constructed using single-copy sequences showed that the tobacco isolate 3T-1 was not classified into the TB subgroup, but was divided into a branch with the PT subgroup and was independent of the PT subgroup, which was consistent with our previous study and confirmed the unique taxonomic status of the tobacco isolate. Furthermore, to explore the differences in virulence factors among the three subgroups, we performed whole-genome and comparative genome analyses showed that the genomes encoded a series of genes related to virulence, with some inter-genomic differences. Our study lays a foundation for exploring the pathogenicity process and mechanism of *Rhizoctonia solani* AG-3 infestation of its hosts.

## 2 Materials and methods

### 2.1 Fungal strain culture and genome sequencing

Mycelial tip cakes were taken and incubated on potato dextrose broth medium at 28°C with 100 rpm shaking for 7 days, fresh mycelium was harvested and ground into powder after freeze-drying. Total DNA of 3T-1 and MJ-102 were isolated from the mycelia using the EZNA Fungal DNA Kit (Tiangen, Beijing, China) following the manufacturer’s instructions. The genome was sequenced by the Illumina and Pacbio Sequel II at Biomarker Technologies (Beijing, China).

### 2.2 Validation of sequencing results

To verify the accuracy of sequencing results and protein-coding gene prediction results, we used TransZol Up Plus RNA Kit (Tiangen Biotech Co., Ltd., Beijing, China) to extract RNA from the sequenced strains. The extracted RNA was reverse transcribed into cDNA using PrimeScript TM RT reagent Kit with gDNA Eraser (Takara Bio Inc., Shiga, Japan). Three genes were selected and primers (Table 1) were designed using Primer 5.0 for PCR amplification. The DNA purification and recovery kit (Tiangen Biotech Co., Ltd., Beijing, China) was used to recover the bands and ligated to the pEASY-Blunt Zero Cloing Vector, and the positive clones were screened for sequencing.

**Table 1:** Primer used in this study.

Primer name	Forward Primer Sequence (5′-3′)	Reverse Primer Sequence (5′-3′)
RS24	ATGAAGTTCTCCGCAATCGT	TTAGAGGCACTGTGAGTACC
RS62	ATGCGCTCTACGACCACTTA	TTACTGGTTCAGCCCCATCC
RS86	ATGAAGTCTGTTTTTGCGCT	TATATCTCAGCCGGACCAGA
RS60	ATGAATTTCAGCACAGCAAC	TTAGTATTCCCTTTGCGACC
RS95	ATGTTGTTTGCTTCTCTCGTC	TTACTTGCGAACACCAAGG
RS91	ATGAGGACCTTCGGAATTGTC	CTAGCAAGCACCGTCATC

### 2.3 Genome assembly and annotation

The ccs reads were assembled by Hifiasm v0.16.0 (Cheng et al., 2021), and the draft genome was corrected after assembly by Pilon v1.17 (Walker et al., 2014). Genscan version 2009-11 (Burge and Karlin, 1997), Augustus v2.4 (Stanke and Waack, 2003), GlimmerHMM v3.0.4 (Majoros et al., 2004), GeneID v1.4 (Blanco et al., 2007), SNAP version 2006-07-28 (Korf, 2004) were used to make *ab initio* predictions. Then, GeMoMa v1.3.1 (Keilwagen et al., 2016) was used for prediction based on homologous proteins. Finally, EVM v1.1.1 (Haas B. J. et al., 2008) was used to integrate the above two methods to obtain protein-coding genes. The predicted proteins were blasted (e-value: 1e-5) against Nr, Swiss-Prot, TrEMBL, KEGG, and KOG. Blast2go (Conesa et al., 2005) was used for GO annotation. HMMER 3.0 (Eddy, 1998) was used for Pfam annotation.

### 2.4 Comparative genome analysis

To comprehensively understand the distinctions between 3T-1, MJ-102, and Rhs1AP (GenBank accession number: JATN00000000), we conducted a comparative investigation of their genome content and genome structure. Family clustering of predicted protein sequences from sequenced strains and protein sequences from the reference genome was performed using OrthoMCL version 2022-08-12 (Li et al., 2003), followed by analysis of gene families. Based on the results of the above gene family clustering analysis, gene families from which both sequenced species and reference species are single-copy genes were identified, and the phylogenetic tree was constructed using PhyML v3.0 (Guindon et al., 2010) to study the evolutionary relationship between species. The predicted protein sequences were compared with the protein sequences of the reference genome by BLAST, and then the software MCScanX v1.1.11 (Wang et al., 2012) was used to draw the collinearity map according to the collinearity relationship.

### 2.5 Predictive analysis of pathogenicity-related gene

Carbohydrate-active enzymes, cytochrome P450, and host-interacting genes researched by Blast against CAZy, CYPED, and PHI databases. antiSMASH v6.0.0 with a web server (Blin et al., 2021) was used to identify probable secondary metabolite biosynthesis gene clusters in the genomes of 3T-1, MJ-102, and Rhs1AP. Effector protein predicted by SignalP 5.0, ProtComp 9.0, TMHMM 2.0, big-PI Fungal Predictor, and EffectorP 3.0 (Xu et al., 2023b).

## 3 Results

### 3.1 Genome sequencing and assembly

**Figure 1:**
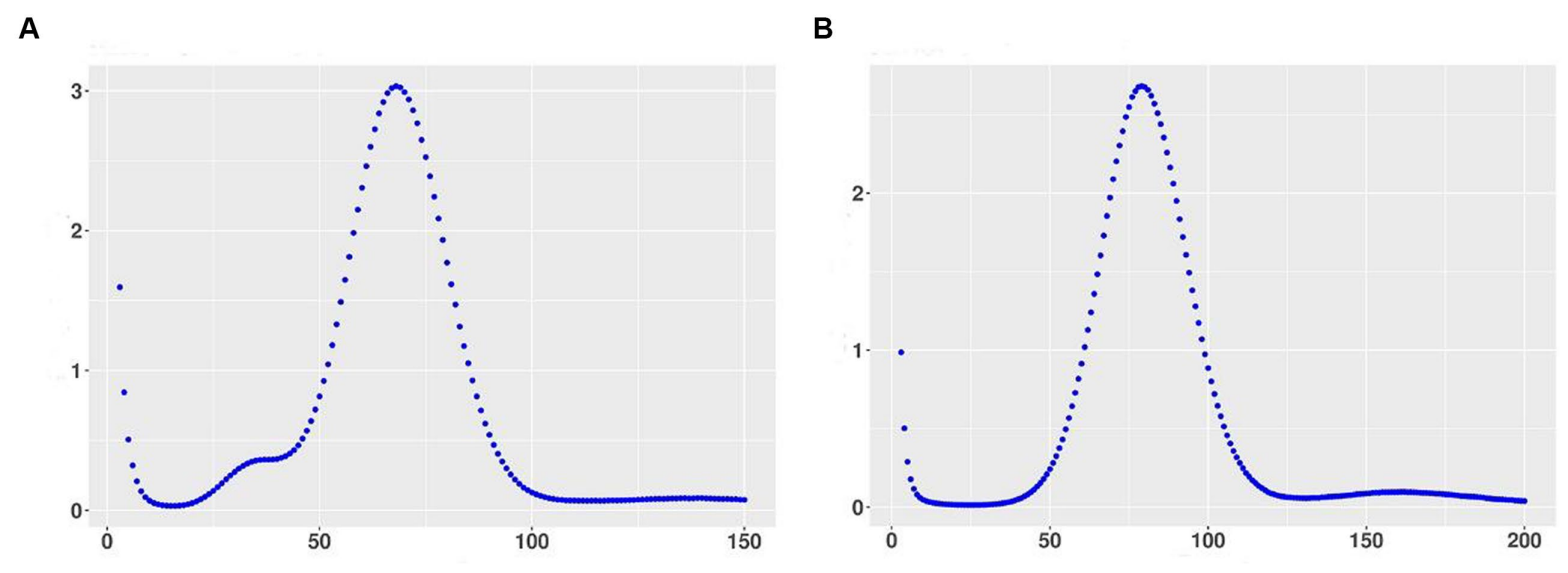
Kmer distribution map with k = 17 using 350 bp library data. **(A)** Kmer distribution of 3T-1; **(B)** Kmer distribution of MJ-102. The X-axis represents sequencing depth, and the Y-axis represents the frequency of k-mer.

The genome of 3T-1 and MJ-102 were assembled from the data generated by the Illumina and PacBio sequencing platforms. A 350 bp library was constructed from the genomic DNA of 3T-1 and MJ-102 samples, and 3.53 and 4.32 Gb of high-quality data were sequenced and filtered on the Illumina NovaSeq sequencing platform, with a total sequencing depth of about 88× and 96 ×, and the Q20 proportion of the sequencing data was all above 97.99% and 98.59%, and the Q30 proportion of the sequencing data was all above 89.45% and 91.96%, respectively. The coverage of the assembled genome was 99.79% and 99.75%. The genome integrity was 95.52% and 94.83% evaluated by BUSCO. K-mer analysis confirmed the high quality of the library (Figures 1A,B). Next, whole genome ccs sequencing was performed by Pacbio Sequel II, 3T-1 sequencing yielded 2.817957533 G subreads data, 2.817957533 G ccs reads data was obtained after processing subreads, 2.438436805 G subreads data was obtained by MJ-102 sequencing yielded, 2.438436805 G ccs reads data after processing subreads. The assembled 3T-1 and MJ-102 genome consisted of 153 and 93 contigs with an N50 length of 2,363,290 bp and 2,529,571 bp, a combined size of 42,103,587 and 41,908,281 bp. The average GC ratios were 49.74% and 48.91% and the repeat rate was 12.08% and 14.98%, respectively. A total of 11,290 and 10,592 protein-coding genes were predicted with a total length of 24,505,659 bp and 23,527,863 bp (Table 2). And the Rhs1AP genome consists of 10,166 contigs, with a N50 length of 8,499 bp, a combination size of 46,445,294 bp, a GC content of 48.23%, and a repeat sequence ratio of 0.78%. A total of 8,954 proteome-coding genes were predicted. It can be seen that the sequenced genome has similar size and GC content to the reference genome, but the sequenced genome obtained fewer contigs and longer N50 length, indicating that the two sequenced genomes have better continuity of assembly, fewer fragments, and higher assembly quality. Sequenced genomes have obtained a larger proportion of repetitive sequences. Repetitive sequences are an important part of gene regulatory networks, which play an indispensable role in gene expression and transcriptional regulation while affecting the evolution, inheritance and variation of life (Ai, 2008).

**Table 2:** Genomic features of 3T-1, MJ-102, and Rhs1AP.

Parameters	3T-1	MJ-102	Rhs1AP
Sequence reads number	293,241	288,980	-
Summary reads base (Gb)	2.81	2.43	-
Coverage (%)	99.79	99.75	-
Contig number	153	93	10,166
Contig length (bp)	42,103,587	41,908,281	46,445,294
Contig N50 (bp)	2,363,290	2,529,571	8,499
GC (%)	49.74	48.91	48.23
BUSCO	95.52	94.83	90.30
Repetitive genome (%)	12.08	14.98	0.78
Coding proteins	11,290	10,592	8,954
Signal protein	1,204	1,096	1,198
Extracellular protein	569	525	625
TMHMM protein	559	512	619
Secretion protein	522	478	579
Effector protein	245	229	278

### 3.2 Detection of protein-coding genes in sequenced genomes

**Figure 2:**
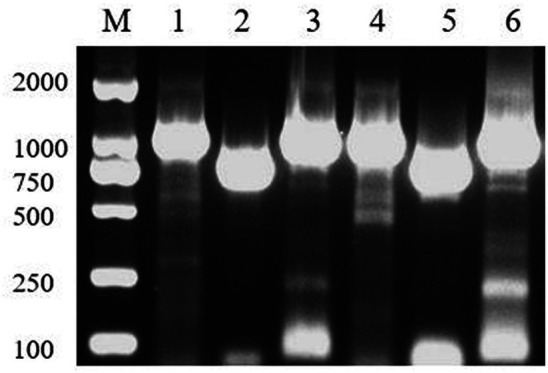
Sequencing genome protein coding gene detection. M, Marker; 1, RS24; 2, RS86; 3, RS62; 4, RS60; 5, RS91; 6, RS95.

The results of PCR detection (Figure 2) showed that the band size was in line with the expected size, and the sequencing sequence was consistent with the whole genome sequencing and prediction results (Supplementary material), indicating that the whole genome sequencing and gene prediction results were reliable.

### 3.3 Gene prediction and annotation

**Figure 3:**
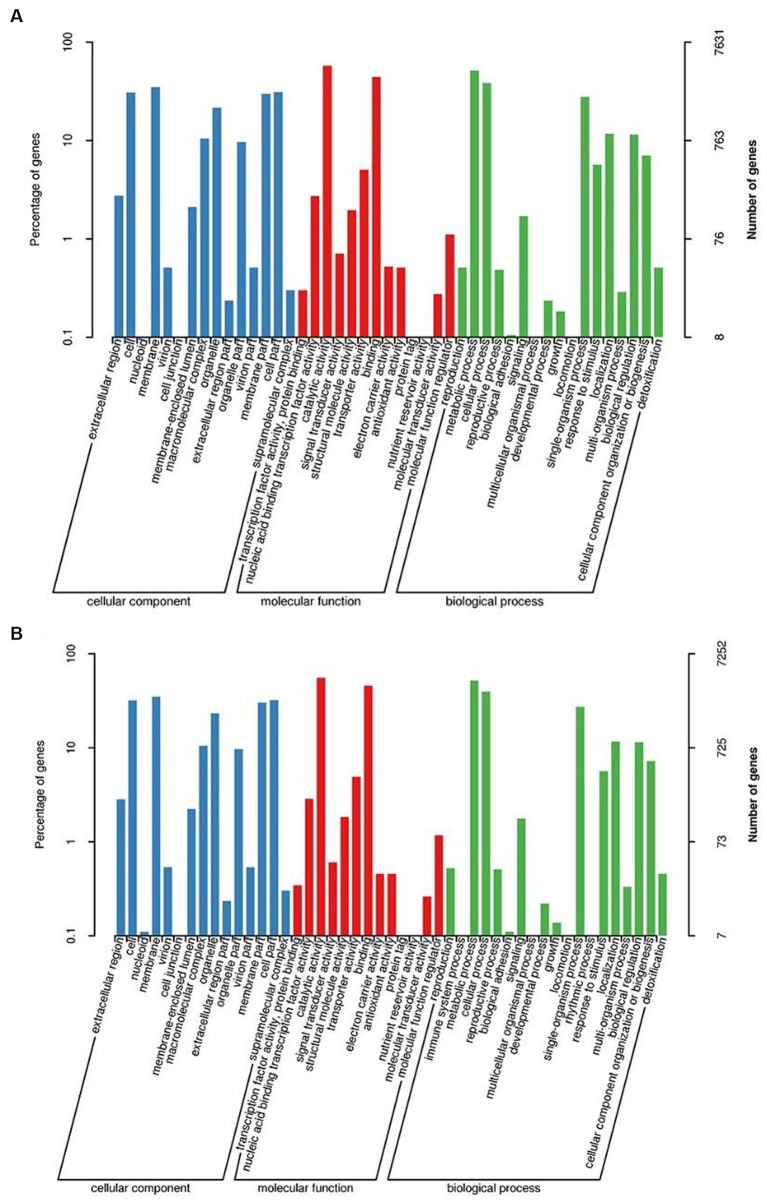

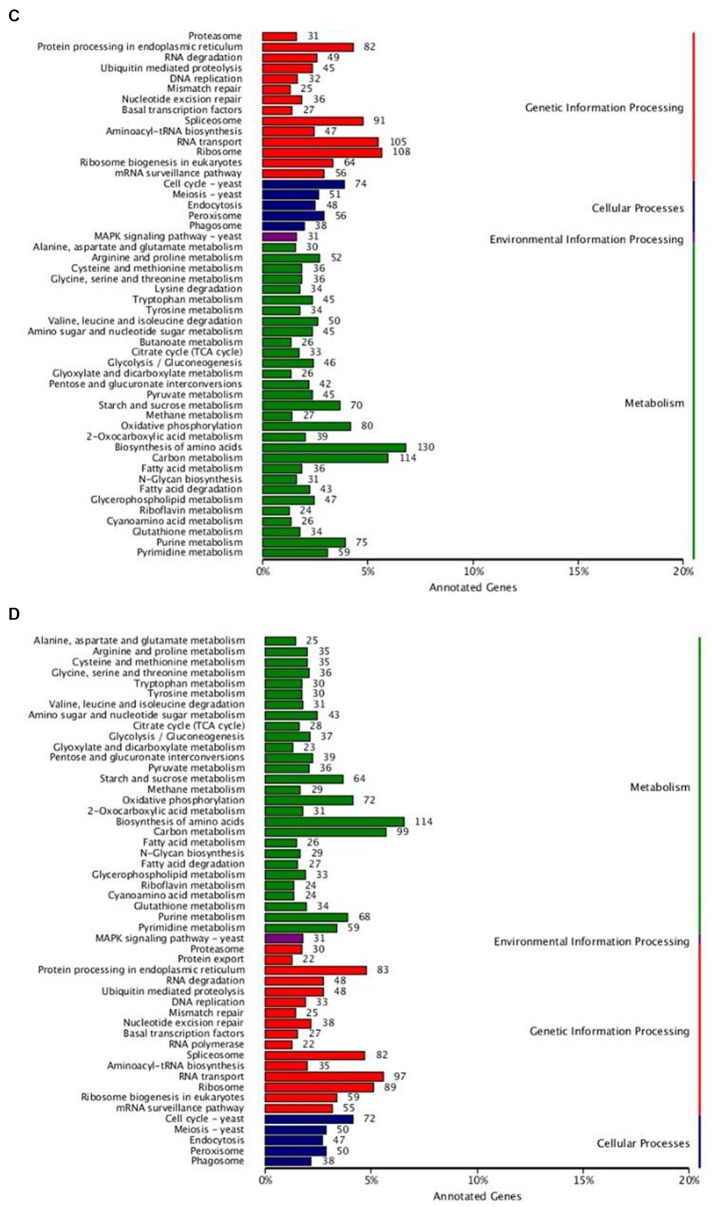
GO and KEGG functional annotation classification of 3T-1 and MJ-102. **(A)** GO annotation of 3T-1. **(B)** GO annotation of MJ-102. **(C)** KEGG annotation of 3T-1. **(D)** KEGG annotation of MJ-102.

GO and KEGG databases were used to analyze the functional classification of 3T-1 and MJ-102. The results (Figures 3A,B) showed that the GO classification annotation was divided into three categories: molecular function, cellular component, and biological process. A total of 7,631 genes in strain 3T-1 were annotated to 45 subclasses, and 7,252 genes in strain MJ-102 were annotated to 47 subclasses. Further analysis showed that 4,459 and 4,271 genes were annotated to molecular function (mainly including catalytic activity, binding, transporter activity, and nucleic acid binding transcription factor activity) in strain 3T-1 and MJ-102, respectively. 2,000 and 1,903 genes were annotated to cellular components (mainly including cell, cell part, membrane, and membrane part), and 1,172 and 1,078 genes were involved in biological processes (mainly including metabolic process, cellular process, and single-organism process).

The KEGG pathway annotation results (Figures 3C,D) showed that a total of 3,249 genes of strain 3T-1 were annotated to 109 pathways, and a total of 3,015 genes of strain MJ-102 were annotated to 108 pathways. The results showed that the pathways mainly included Metabolism, Genetic information processing, Environmental information processing, and Cellular processes. In the Metabolism category, Biosynthesis of amino acids (ko01230) had the most annotated genes, with 130 and 114 genes in strains 3T-1 and MJ-102, respectively, followed by 114 and 99 genes annotated to Carbon metabolism (ko01200). In the genetic information processing category, 108 and 89 genes were annotated to the ribosome (ko03010) pathway, and 105 and 97 genes were annotated to the RNA transport (ko03013) pathway, respectively. The only annotation in the Environmental information processing category is the MAPK signaling pathway-yeast (ko04011) pathway, which also has 31 genes in 3T-1 and MJ-102. The cell cycle-yeast (ko04111) pathway was more annotated in cellular processes, with 74 and 72 genes, respectively.

### 3.4 Comparative genomics

**Figure 4:**
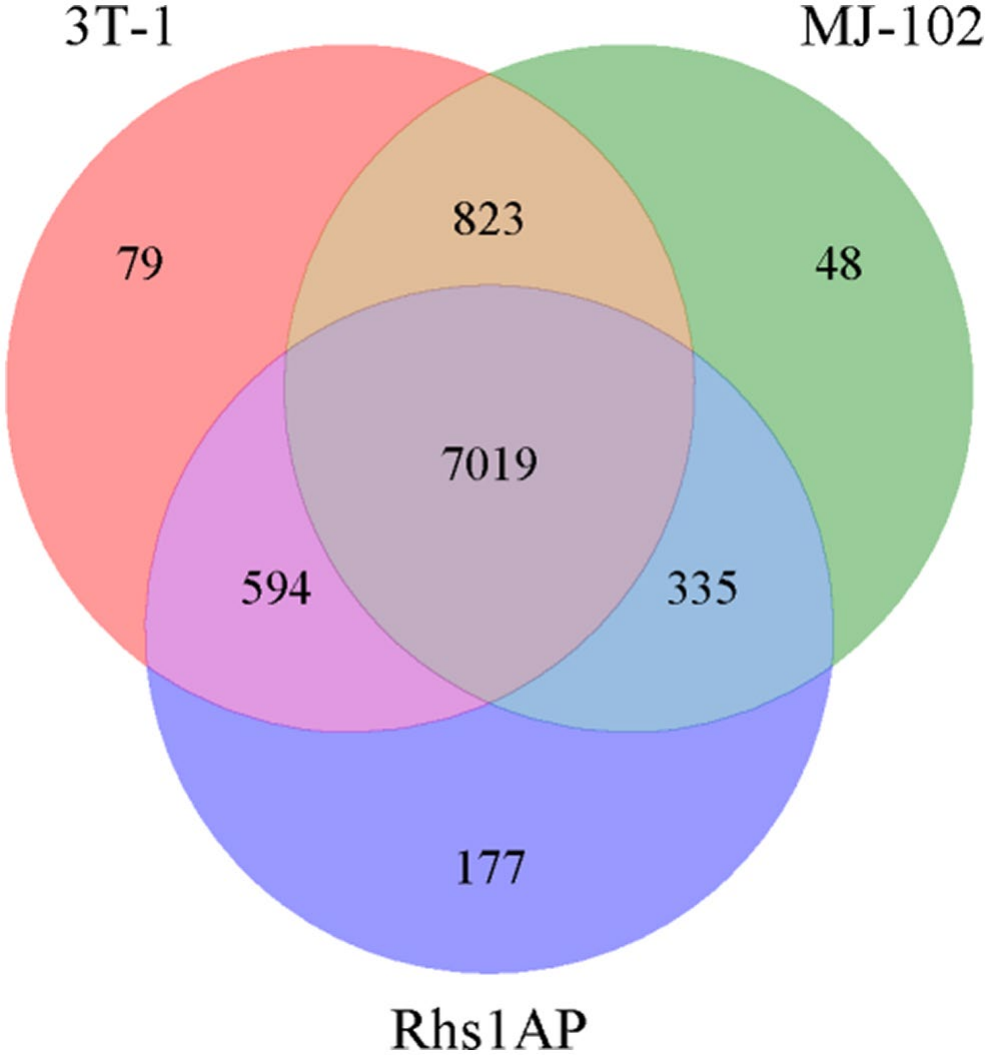
Interspecies gene family Venn diagram. The letters represent the names of the three strains, and the data in the Venn diagram represent the number of conserved and non-conserved sequences between the three fungal species.

The gene families of the sequenced and reference strains were analyzed, including the statistical analysis of strain-specific gene families, gene families shared by strains, and gene families that were single-copy in each strain, and the Venn diagram (Figure 4) was constructed for the statistical results of the gene families, and the gene families were functionally annotated based on the Pfam database. The specific statistical information is shown in Supplementary Table S1. There are 7,019 gene families common to 3T-1, MJ-102, and Rhs1AP, 79 gene families unique to 3T-1 compared to MJ-102 and Rhs1AP, 48 gene families unique to MJ-102 compared to 3T-1 and Rhs1AP, and 177 gene families unique to Rhs1AP compared to 3T-1 and MJ-102. These unique family genes may be related to the specificity of the species.

### 3.5 Phylogenetic analysis of *Rhizoctonia solani*

**Figure 5:**
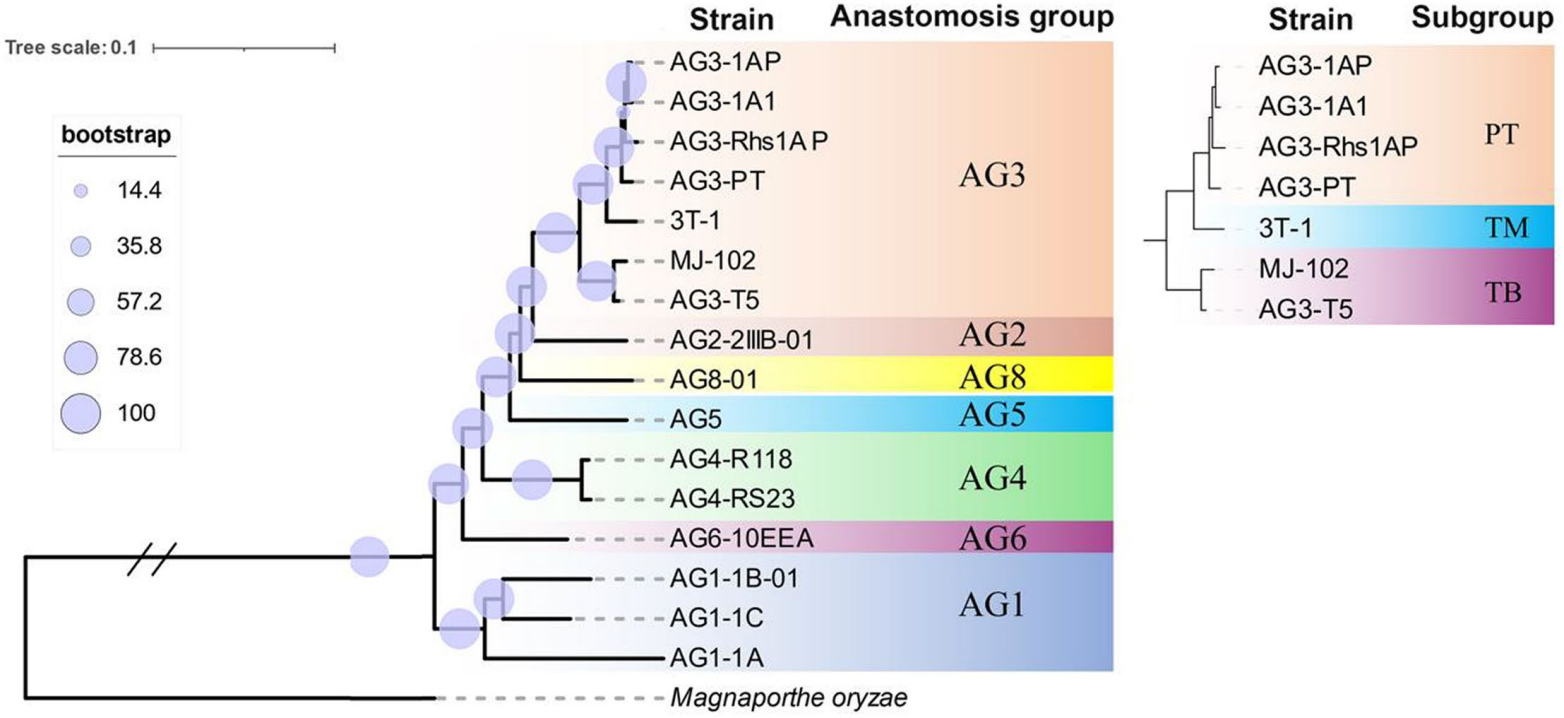
Phylogenetic tree of different anastomosis groups and subgroups.

The single-copy genes of 14 strains in seven reported fusion groups and two strains sequenced in this study were selected to construct the phylogenetic tree. The results showed that the same fusion group and its subgroups were clustered together. In the branch of AG-3, the TB subgroup and the PT subgroup were divided into two branches, while the strains 3T-1 and PT were classified into one branch and independent of other PT strains (Figure 5). This result is consistent with the phylogenetic tree constructed by the ITS sequence in our previous report (Xu et al., 2023a).

### 3.6 Genomic collinearity analysis

**Figure 6:**
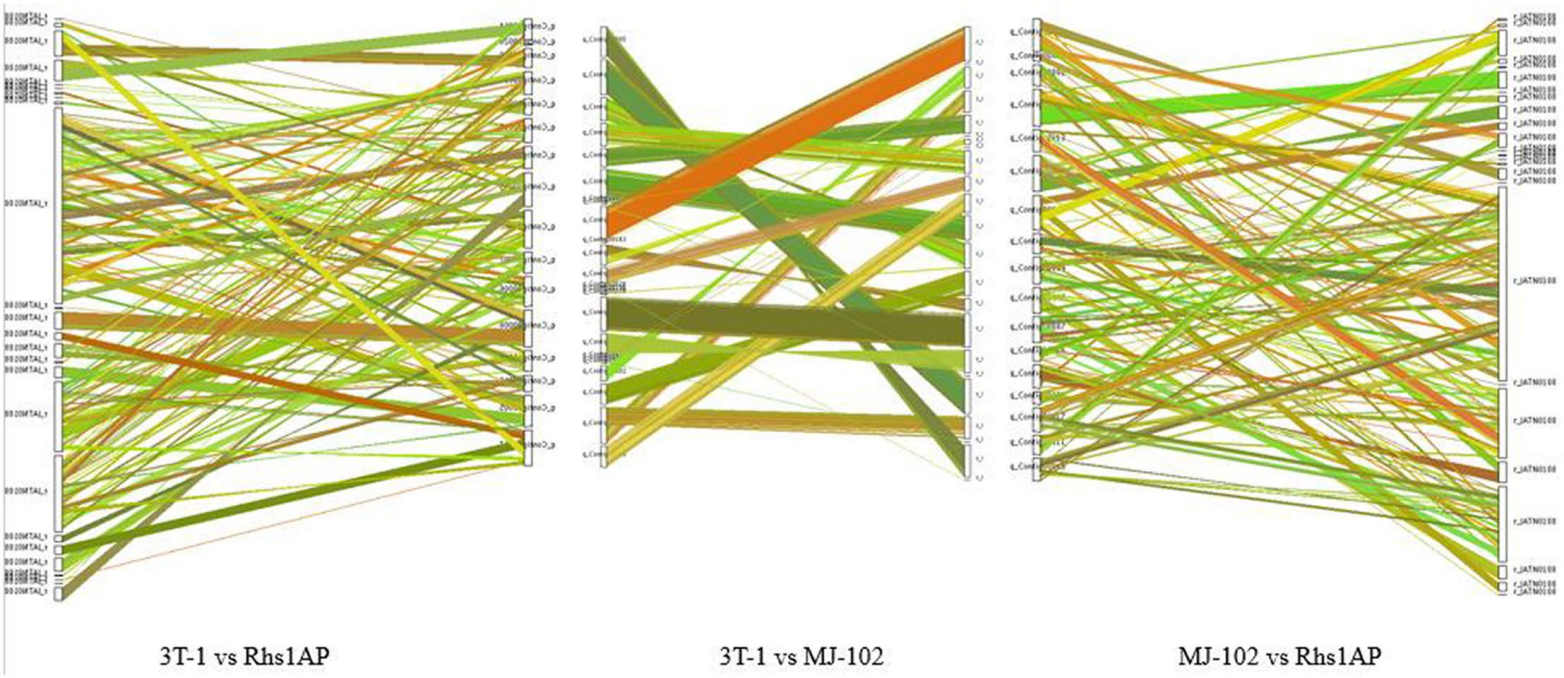
Genomic collinearity analysis. The color of the line represents different comparison results; the thickness of the line indicates the degree of collinearity.

There are a large number of genomic structural variations during the evolutionary process, which lead to some coding gene function changes, and may even lead to some functional protein changes, especially in regions of non-collinearity comparison, which are more likely to be associated with virulence differences. The genomes of sequenced strains and reference strains have some collinearity, but there are also insertions, deletions, inversions, and translocations of sequences (Figure 6). Among them, the degree of collinearity between 3T-1 and MJ-102 was much higher than that between 3T-1 and Rhs1AP and MJ-102 and Rhs1AP.

### 3.7 Pathogenicity-related genes in the genome of 3T-1, MJ-102, and Rhs1AP

Many known or putatively linked genes encoding virulence factors were found in the genome of 3T-1, MJ-102, and Rhs1AP. These virulence factors encode the genes related to carbohydrate-active enzymes (CAZymes), Cytochrome P450s, gene clusters for secondary metabolites, genes of pathogen-host interactions, and effector proteins (Supplementary Table S1). By comparing the pathogenicity-related genes of 3T-1, MJ-102, and Rhs1AP, we found that there are many similarities among the three, but there are also some differences. For example, GH24, GT26, and PL6 in carbohydrate-active enzymes are unique to 3T-1, while PL22 is unique to MJ-102.

### 3.8 Carbohydrate-active enzymes database annotation

**Figure 7:**
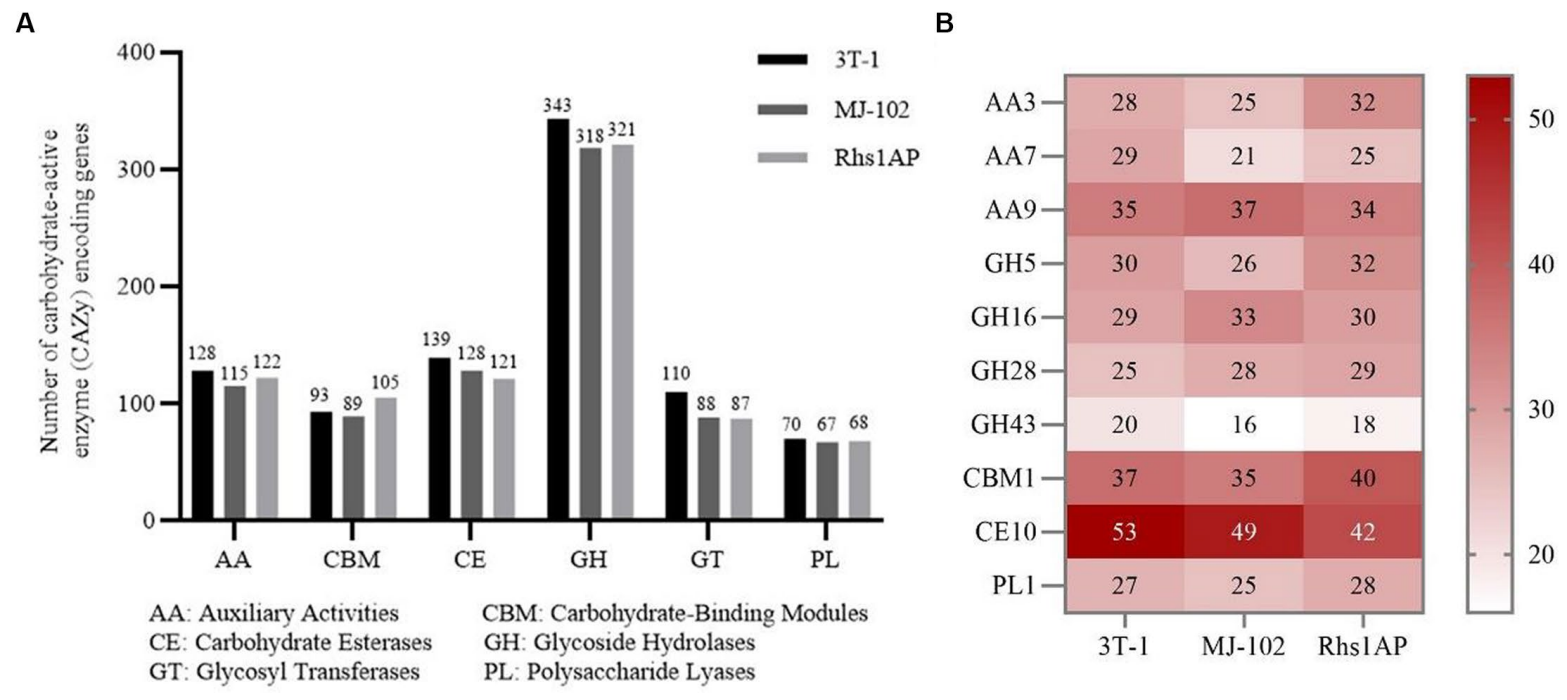
Analysis of different CAZyme families in 3T-1 and MJ-102. **(A)** Total number and category of CAZymes in the predicted secretome. **(B)** Comparative analysis of important fungal and plant cell wall degrading CAZyme families in the genomes of 3T-1 and MJ-102.

Cell wall-degrading enzymes play an important role in the pathogenesis of plant pathogens (Yang M. et al., 2012). There are a large number of cell wall degrading enzymes 760, 692, and 747 in strains 3T-1, MJ-102, and Rhs1AP, respectively. These cell wall degrading enzymes are mainly divided into six categories: Auxiliary Activities (AA), Carbohydrate-binding modules (CBM), Carbohydrate Esterases (CE), Glycoside Hydrolases (GH), Glycosyl Transferases (GT), Polysaccharide Lyases (PL). Among them, Glycoside Hydrolases are the main category of cell wall degrading enzymes in strains 3T-1, MJ-102, and Rhs1AP, and compared with MJ-102 (GHs: 318 genes) and Rhs1AP (GHs: 321 genes), strain 3T-1 has a larger number of Glycoside Hydrolases (GHs: 343 genes), followed by Carbohydrate Esterases, Auxiliary Activities, Carbohydrate-binding Module, Glycosyl Transferases, and Polysaccharide Lyases (Figure 7A). Further analysis showed that CE10, CBM1, AA9, AA3, AA7, GH5, GH16, GH28, GH43 and PL1 were more annotated, among which CE10 annotated the most genes, especially in strain 3T-1 (53) (Figure 7B).

### 3.9 Cytochrome P450s

**Figure 8:**
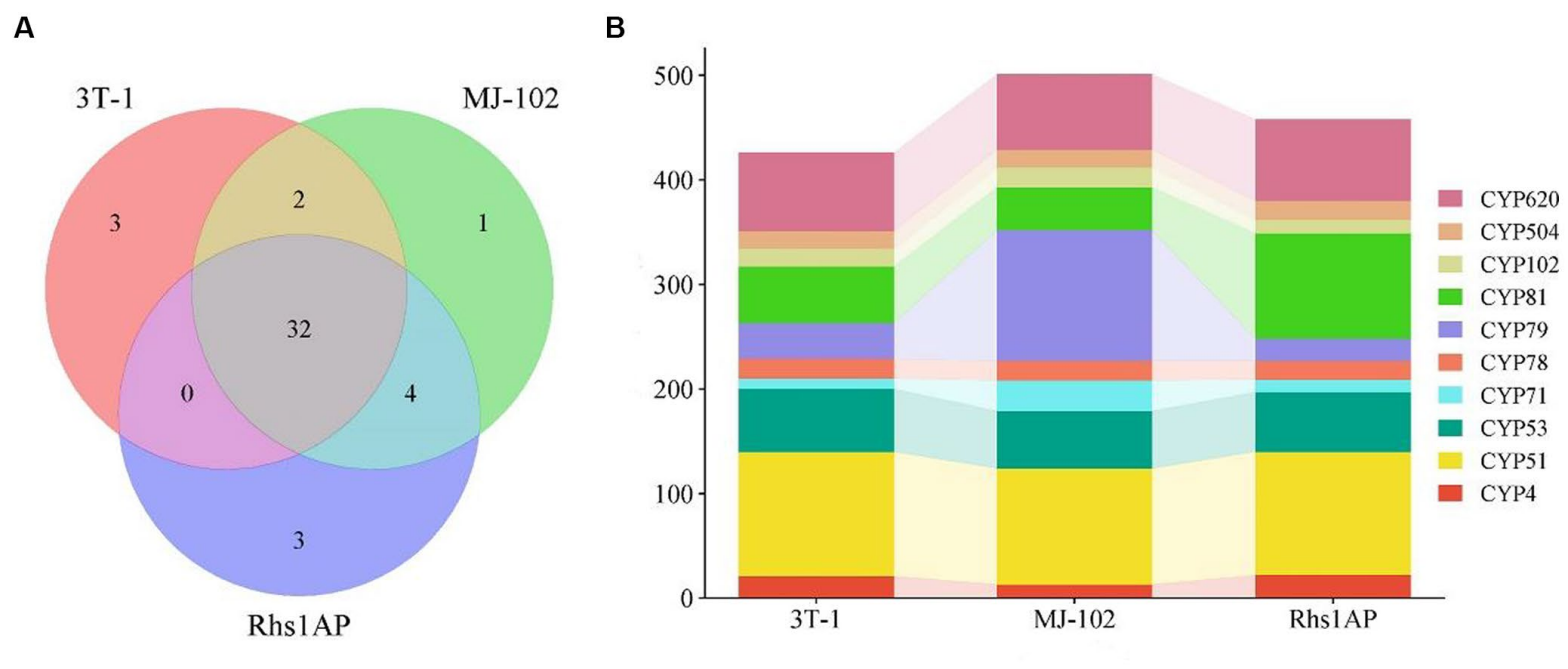
Number of 3T-1, MJ-102, and Rhs1AP annotated to cytochrome P450. **(A)** Veen diagram represented the number of conserved and non-conserved sequences between the three fungal species. **(B)** Annotation to major cytochrome P450 families. The vertical axis represents the number of genes annotated as this family.

Cytochrome P450 plays an important role in the synthesis of fungal secondary metabolites and the metabolism of foreign compounds (Park et al., 2008). In this study, 539, 621, and 556 genes were annotated to 45 cytochrome P450 families in 3T-1 (37), MJ-102 (39), and Rhs1AP (39), respectively. Among them, 32 cytochrome P450s were shared by the three strains, three (CYP92, CYP105, and CYP187) unique to 3T-1, one (CYP109) unique to MJ-102, and three (CYP108, CYP512, and CYP709) unique cytochrome P450 types for Rhs1AP (Figure 8A). In addition, there were also some differences in the number of genes annotated to various cytochrome P450 families in strains 3T-1, MJ-102, and Rhs1AP. We analyzed the top 10 cytochromes P450 families, and the results showed that the number of CYP79 annotated in MJ-102 was much higher than that in 3T-1 and Rhs1AP, and the difference was the largest, followed by CYP81, which was the most abundant in Rhs1AP, the remaining difference is not significant (Figure 8B).

### 3.10 Prediction of gene clusters for secondary metabolites

**Figure 9:**
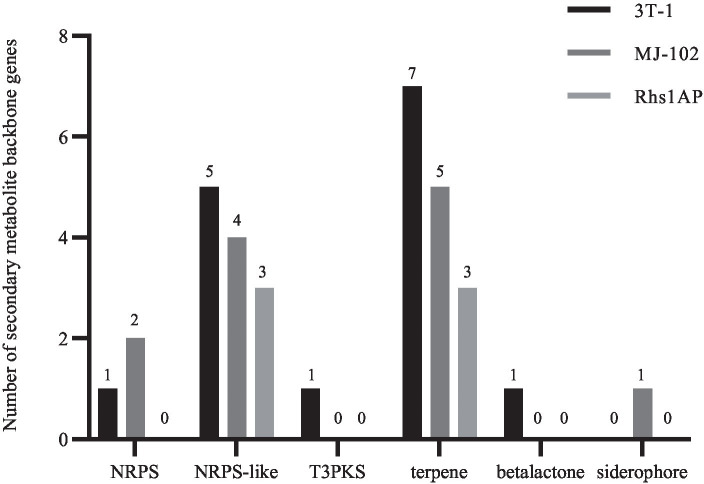
Prediction of secondary metabolite synthesis gene clusters.

Studying the composition of the secondary metabolite gene cluster of pathogens and the function of related genes is helpful to the research progress in the field of pathogenic mechanisms (Li Y. J. et al., 2019). Through prediction and analysis in antiSMASH software, the results (Figure 9) showed that 15, 12, and 6 secondary metabolic gene clusters were predicted in the genomes of 3T-1, MJ-102, and Rhs1AP, respectively. Among them, one t3PKS gene cluster, one NRPS gene cluster, five NRPS-like gene clusters, seven terpene gene clusters, and one beta lactone gene cluster were predicted in 3T-1, two NRPS gene clusters, four NRPS-like gene clusters, five terpene gene clusters, and one siderophore gene cluster were predicted in MJ-102, while only three NRPS-like gene clusters and three terpene gene clusters were predicted in Rhs1AP.

### 3.11 Pathogen-host interactions database annotation

PHI (Pathogen Host Interaction Database), which contains pathogenicity genes, virulence genes, and effector protein genes of bacteria, fungi, and other pathogens that have been experimentally verified or reported in the literature to be able to infect plants, animals, fungi, and insects. In addition, antifungal compounds and their target genes are also included (Winnenburg et al., 2006). Genome-wide annotation of 3T-1, MJ-102, and Rhs1AP using the PHI database resulted (Table 3) in the annotation of 3,093, 2,983, and 3,132 genes, respectively. In 3T-1, MJ-102 and Rhs1AP, 284, 256, and 279 genes were annotated with loss of pathogenicity, 156, 135, and 125 genes were annotated with increased virulence, 1,389, 1,433, and 1,381 genes were annotated with reduced virulence, 7, 5, and 6 genes were annotated with effector, and 16, 15, and 15 genes were annotated with chemical target loci (chemistry_target).

**Table 3:** PHI database annotation result statistics.

Phenotypic category	3T-1	MJ-102	Rhs1AP
	Number of gene PHI	Number of gene PHI	Number of gene PHI
Reduced virulence	1,389	1,433	1,381
Unaffected pathogenicity	846	777	900
Increased virulence	156	135	125
Lethal	108	107	116
Loss of pathogenicity	284	256	279
Mixed outcome	287	255	310
Effector (plant avirulence determinant)	7	5	6
Chemistry target	16	15	15
Total	3,093	2,983	3,132

### 3.12 Prediction of secretory and effector proteins

Effector proteins play an important role in the pathogenic process of pathogenic fungus, using the software SignalP 5.0 to predict and analyze all the protein sequences. The total number of proteins predicted to contain signal peptides in 3T-1, MJ-102, and Rhs1AP were 1,204, 1,096, and 1,198 of which 569, 525, and 625 were predicted to be secreted into the extracellular region, and among the proteins secreted into the extracellular region, 559, 512, and 619 proteins were predicted to contain 0–1 transmembrane structural domains, among them, 522, 478, and 579 proteins did not contain GPI anchor sites, that is, 522, 478, and 579 proteins in 3T-1, MJ-102. Further, 245, 229, and 278 effector proteins in 3T-1, MJ-102, and Rhs1AP were predicted by EffectorP (Table 2).

## 4 Discussion

*Rhizoctonia solani* has a wide range of hosts and has the characteristics of multi-core, heterozygosity, and sexual reproduction degradation, which makes it difficult to obtain high-quality genome sequences (Zheng et al., 2013). Sequencing of related *R. solani* AG-3 strains can lay the foundation for comparative genomics studies exploring the pathogenicity correlates of these organisms (Cubeta et al., 2014; Wibberg et al., 2017; Kaushik et al., 2022). Recent advances in long-read sequencing technologies have led to significant improvements in the quality of genome assemblies, particularly due to the merging of contigs and scaffolds that span gaps around repeat regions (Lee et al., 2021). Previously, *R. solani* was sequenced using a variety of sequencing methods, but the N50 length of its contigs was not comparable to other sequenced genomes. We performed comparative genomics analysis of whole genome sequencing of *R. solani* AG-3 strains from two regions using Illumina HiSeq short-read in combination with PacBio long-read sequencing, and obtained N50 lengths of 2,363,290 and 2,529,571 bp, with an average *de novo* genome assembly size of about 40 Mbp. This genome size is comparable to that of the previously sequenced reference genome (Cubeta et al., 2014), but is different from the size of sequence AG3-1A1 (71 Mb; Kaushik et al., 2022) from the long-read sequence isolate. There are more repetitive regions in our genome assembly than in the reference genome. The proportion of tandem repeats in the genome is different in different organisms, and many repeats have significant biological functions in the genome. For example, repeated sequences located in the protein-coding region, if expanded or shrunk, will cause genes to lose function or regain new functions due to frameshift mutations or prolonged harmful mRNA production, and ultimately all these effects can lead to changes in biological phenotypes (Li et al., 2004). In this study, 3T-1 has more types of repetitive sequences, while MJ-102 has more repetitive sequences. Among them, MJ-102 contains the most LTR/Gypsy repetitive sequences, and the function of related repetitive sequences remains to be further studied.

There are large differences in morphology and pathogenicity among different isolates of *R. solani* AG-3, and the wide distribution of strains with different levels of aggressiveness affects the breeding and deployment of resistant varieties of host crops (Su et al., 2016). However, genomic comparisons have not yet been performed. Previously, 69 tobacco and potato isolates from Yunnan and Liaoning regions of China were evaluated for virulence and aggressiveness against their hosts, and significant differences in virulence and aggressiveness of isolates on the host. We revealed significant differences in the level of aggressiveness of different strains based on leaf and rhizome inoculation tests. The growth rate of colony formation on the PDA medium and areas of damage on inoculated isolated leaves showed differences in the level of aggressiveness of different strains on the host. Based on these observations, we categorized them as low, moderate, and highly aggressive isolates. Both 3T-1 and MJ-102 are highly aggressive isolates, and both 3T-1 and MJ-102 are tobacco isolates, yet 3T-1 is classified in the AG-3TM subgroup in the phylogenetic tree constructed from ITS sequences (Xu et al., 2023a). Similarly, in this study, although 3T-1 was isolated from tobacco, the phylogenetic tree constructed by single-copy sequence showed that 3T-1 and PT subgroups were clustered into one branch, independent of PT subgroup, belonging to the branch of TM subgroup, and TB subgroup was another branch, which once again confirmed the unique taxonomic status of 3T-1, and also showed that there were some differences between TM subgroup and TB and PT subgroup, and there were also some correlations.

The virulence factors of fungi usually include cell wall degrading enzymes, secondary metabolite gene clusters, and effector proteins (Carling et al., 2002). In this study, we found a large number of glycoside hydrolases (GHs) in 3T-1 and MJ-102. GHs can cleave the glycosidic bonds of polysaccharide components in the cell wall (Kubicek et al., 2014), so we believe that they secrete a large number of glycoside hydrolases to decompose the plant cell wall, thereby helping the infection. In this process, some cell wall degrading enzymes of CE10, CBM1, AA9, AA3, AA7, GH5, GH16, GH28, GH43, and PL1 families were annotated. Among them, AA3 and AA7 have some functions related to ligninolytic enzymes, AA9 and GH16 have cellulase activity, and GH28, GH43, and PL1 have pectinase activity (Roy et al., 2020). They all play a certain role in the process of infecting the host, and the difference in the number and type of cell wall degrading enzymes in different strains may be one of the reasons for the difference in pathogenicity.

Cytochrome P450 is known to play an important role in the synthesis of fungal secondary metabolites and the metabolism of foreign compounds (Da Silva et al., 2003). In this study, more genes were annotated to CYP4, CYP51, CYP53, CYP71, CYP78, CYP79, CYP81, CYP620. Among them, compared with 3T-1 and Rhs1 AP, more genes were annotated to CYP71 and CYP79 in strain MJ-102. CYP71 could enhance the resistance of rice blast (Li et al., 2014). CYP79 has been reported to play an important role in biosynthesis and plant growth and development (Chen et al., 2010). Compared with 3T-1 and MJ-102, Rhs1AP has more gene annotations to CYP81. CYP81 has been reported to enhance the resistance of Arabidopsis and tobacco to herbicide pesticides (Liu et al., 2012). Therefore, we believe that these different cytochrome P450 family members may play a role in resistance to pathogens and inhibition of host resistance. In addition, there are some unique cytochrome P450s, among which CYP92, CYP105, and CYP187 are unique to 3T-1. It has been reported that CYP92 expressed in petunia flower buds can catalyze the monooxidation of long-chain fatty acids (Petkova-Andonova et al., 2002). *Streptomyces griseus* CYP105A1 catalyzes the hydroxylation of vitamin D (Sugimoto et al., 2008). The function of CYP187 has not been reported. CYP109 unique to MJ-102 was reported to be the vitamin D3 25-hydroxylase of *Bacillus megaterium* (Abdulmughni et al., 2017). CYP108, CYP512, and CYP709 are unique to Rhs1AP. Among them, CYP108D1 of *Novosphingobium aromaticivorans* DSM12444 is an aromatic hydrocarbon binding P450 enzyme (Bell et al., 2012). CYP709C1 can catalyze the sub-terminal hydroxylation of C18 fatty acids (Kandel et al., 2005). The function of CYP512 has not been reported.

A certain number of NRPS gene clusters were found in 3T-1 and MJ-102, but not in Rhs1AP. It was found that all known fungal siderophores were synthesized by NRPS (Haas H. et al., 2008), which proved that there was a certain ability to absorb iron in 3T-1 and MJ-102. Therefore, reducing the bioavailability of plant pathogens to Fe^2+^, reducing the content of available iron in the environment, and reducing the pathogenicity of pathogens can accelerate the aging and death of pathogens. This finding can provide a target for the prevention and treatment of such diseases. Toxin is an important pathogenic factor of *R. solani* (Yang Y. Q. et al., 2012). The study on the pathogenicity, biosynthesis pathway, and regulatory network of plant pathogenic mycotoxins is of great significance for revealing the infection mechanism, pathogenic mechanism, and pathogen-host interaction of pathogens. The biosynthesis of toxins is generally regulated by the gene cluster where the gene encoding polyketide synthase (PKS) is located (De Jonge et al., 2018). In this study, T3PKS was predicted in 3T-1, which can be used as a potential target for toxin research.

In addition, we found a comparable number of genes encoding potentially secreted and effector proteins in each genome. Several effectors have been reported for the *R. solani* AG-1 fusion cluster (Yang Y. Q. et al., 2012; Zheng et al., 2013; Chen et al., 2018; Li S. et al., 2019; Rao et al., 2019; Wei et al., 2020), whereas *R. solani* AG-3 has yet to be relevantly reported. In terms of the secreted proteins in phytopathogenic fungi and antagonistic bacteria, previous studies have found that the amounts of secreted proteins contained in phytopathogenic fungi, oomycetes, and bacteria are not equal, with the proportion of fungal secreted proteins in the whole protein ranging from 3.65% to 9.58% (Han and Wang, 2017). The proportions of secreted proteins in the whole protein quantity of the two sequenced strains in this study were 4.62% and 4.51%, respectively, which were consistent with the aforementioned proportions of secreted proteins in plant pathogenic fungus. Pathogenicity-associated genes are usually clustered in relatively less conserved, rapidly evolving, and gene-poor genomic regions containing large numbers of transposons or repetitive elements (Haas et al., 2009; Dutheil et al., 2016). These genomic regions typically contain effector genes involved in the evolutionary adaptation of the pathogen to the host (De Jonge et al., 2018). Rapid evolutionary events in these genomic regions can generate segregation-specific or unique non-core genes that can diversify different strains of the same species (Dale et al., 2019). The unique characteristics of secreted proteins, effector proteins, and similar CAZymes may reflect their ecological and host adaptation strategies. There are some differences between secreted proteins and effector proteins as well as some virulence factors in the genomes of the sequenced strains, but what these differences ultimately lead to and whether the differences between them are due to these genes requires further research to better understand the biology and pathology of this species complex.

Gene family clustering analysis helps to identify common and unique homologs among nearly and distantly related pathogenic species and to study their phylogeny and evolutionary dynamics. The number of homologous and unique gene clusters in the two *R. solani* AG-3 strains differed somewhat from the previously reported Rhs1AP genome (Cubeta et al., 2014). To the best of our knowledge, the AG-3 TB subpopulation can produce sexual spores, whereas AG-3 PT does not, and host specialization exists among the strains, likely, the gene family that is shared in 3T-1 and MJ-102 and absent from Rhs1AP is associated with spore production and pathogenicity, including host specialization.

In conclusion, we sequenced and compared the genomes of two strains of tobacco target spot pathogen isolated from two regions, and our analyses suggest some differences in taxonomic status between them. In addition, we detected gene families for pathogenicity-related genes, effectors, and transporter proteins in the isolates. Resequencing of these isolates showed significant differences between these two genomes and with the reference genome Rhs1AP, suggesting some changes in pathogenicity-related genes during evolution. Our results elucidate the genomic similarities and differences among tobacco target spot disease isolates from different regions and lay the foundation for further studies on their genetic evolution as well as genomic and sequence differences in pathogenicity-related genes.

## Data availability statement

The datasets presented in this study can be found in GenBank. The names of the accession numbers are PRJNA1024900 and PRJNA1024910.

## Author contributions

SX: Writing – review & editing, Writing – original draft, Formal analysis, Data curation. CS: Writing – review & editing, Methodology. CL: Writing – review & editing, Supervision, Funding acquisition. WD: Writing – review & editing, Supervision. GY: Writing – review & editing, Supervision, Funding acquisition, Conceptualization.
