# Supplementary material for: Genome sequencing and comparative genome analysis of Rhizoctonia solani AG-3
Source: Front Microbiol. 2024 Apr 4;15:1360524. doi: 10.3389/fmicb.2024.1360524 (PMC11024465; doi:10.3389/fmicb.2024.1360524)
Supplement: Supplementary file 1 [file Table_1.docx]

**Genome sequencing and comparative genome analysis of *Rhizoctonia solani* AG-3**

Shanshan Xu, Chengmeng Shen, Chengyun Li, Wenhan Dong, Genhua Yang^*^

State Key Laboratory for Protection and Utilization of Bio‐Resources in Yunnan, Yunnan Agricultural University, Kunming, Yunnan, China

*Correspondence: Genhua Yang ghyang2003@126.com

Sequence

>RS24

ATGAAGTTCTCCGCAATCGTCGCTCTCCTTGCTTCGGCCTCTGCCGCATATGCTCATACCACTGTCAAGGCTGTTTTCATCAACGGTGCCGACCAGGGTAATGGCGAGAACACCTACATCCGTTCACCCCCCAACAACAACCCAGTCAAGGACTTGTCCTCTGGCGCTGTTGCTTGCAATGTCAACAACCGTGCTGTCTCGAAGACCCTCGAGGTGTCCGGTGGTGATGTGATTACCTTCGAGTTCGCTCATGACAACCGTCACGACGACATTATGGACCCCACCCACAAGGGTCCTGTCATGGTATATGTCGCCCCCACTTCCTCCAACGGCCAGGGTGCTGTCTGGGTCAAGCTCCACCAGGAGGTGTACACCAACGGCCAATGGGCCTCGGACAAGCTCATCGCAAATGGTGGTCATGTCTCTATCACCGTCCCCGACCTCAAGGCCGGCGAATACATCTTCCGCCCCGAGATCGTCACCCTTCATGAGGCCGACACTGCCTACAGCGCCAACCCGGCTCGTGGTGTGCAGCTGTACATGGAGTGCATCCAGTTCAAGGTGGTCTCCTCCGGCTCCGTCTCGCTTCCCGCCGGCATCGACTTCAAGCAGTCGTACACATATGCCGACAAGGGACTGGTGTTCAACCTCTACGGCTCGGACGCCAAGACGTACGTCGCTCCTGGTGGCGCTGTCTCTTCCATCGCTGCCTCCAACCCTGGTATCGGACCTGTCCCCGCAGCCGGTAGCAAGCCCACCACTAAAGCTGCCAGTCAGCCCACCACCACGGCCAAGCCCATCACTACCGCGGCTGCCACGCCCACCAAGATGACCACCGTCACCGTCAAGCCTACCACGACTGCTGTATCCGGCGGTGGATCTAGCAGCGGAACTGTTGCCAAGTATGGTCAATGTGGAGGTAACGGCTACTCCGGCCCTACCCAGTGTGCTTCAGGCTCGACCTGCAAGCTCAACAACGAGTGGTACTCACAGTGCCTCTAA

>RS62

ATGCGCTCTACGACCACTTATCTTGCTTGTCTGGCACTTTTGGTGAAGTCTGCGTCGGCAGTTGCTGTGTGGGGGCAATGCGGGGGCATTGGACACACTGGGTCGACGGTTTGTGATGCAGGGACTACTTGCGTGAAGGTGAACGATTACTATTCCCAATGTCAACCTGGCAGCGCAGCGACCACGGCATCGCCGACTTCGACCAGTTCCACGACGCAACCTACTGGAGGGGCTAATATTCCTCGTGGAGTTTTGACCAAGATCACAAACTTTGGAAGCAACCCCACCAATATAGAAAATTATGTTTACGTTCCCACCACACTCAAATCCAAGCCGGGTCTACTTGCCGCAATCCACTACTGCACTGGAACAGCCCAGGCCTACTACTCGGGCACTCAATACAAGCAGCTCGCCGACCAATACGGCTTTATCGTCTTGTACCCTGATGCTCCTGATAGCGGAGGATGCTGGGACGTCCACAGCACCGAGACACTCACCCACAATGCTGGCGGTGACTCTCTCGGAATCGCCAGCGGTCTTCGATACCTCATCACCCAATACAATGTAGACACCAACAAAGTCTTCGCTACTGGGACGTCTTCCGGAGCAATGATGACCAACGTCCTTGCGGGTGCTTACCCAGACTTGATCCGTGCCGGTGCGGCATTTGCTGGCGTTCCTTATGGCTGCTTCGCCGGTTCTGGCATGTGGAATAGCCAGTGTGCTACCGGTCAACTCATTAAGACTGCTCAACAATGGGGCGACCAGGTTCGCTCTGGTTATCCAGGGTATACCGGCACTCGTCCCAAGATGCAGCTCTGGCACGGCTCTACCGACACTACCCTCTACACGCAGAACTTCTACGAGGAGATTAAGCAGTGGACGAATGTGTTCGGTGTTAGCCAGACCGCTACTGCTACCACTCAAAACTGGCCCCTTTCCGGTTGGACCAAGACGGAATACGGTCCCAACGTCCAAGCTATTATCGCCAGCGGCGTTGATCACAACCTCCCAGTTCAGGCCACTCAGGTGATCCAATGGATGGGGCTGAACCAGTAA

>RS86

ATGAAGTCTGTTTTTGCGCTCGTTGCTCTTGCCGCTGCTGCCATTCAGCCAGCTCTGGCTCACTATCGTTTTAACAAATACATTGACGCTGCAGGCACTGTGACTGGCGAATACGTTTACGTTCGTGCCAACACAAACATCAACTCGCCGCTCACTGATGTTGCCTCCACCGACATTCGATGCAACGCCGGCGGTCTTACATCTGGAAGTAAGACATCGACGGCCACTGTAGCAGCCGGCTCAACTGTTGGCTTTGAGGCTGACATTCCTATGTCTCATCCTGGGCCGATGTTGGTATACCTTGGAAAGGTGCCATCCGGCCAGACCGCCGCGACTTGGGATGGAAGTGGTGCCAACTGGTTCAAGATTCATCAAATTGGTGCTGACTTTAGCACCGGTGCTTTAAAGTGGCCTACAGATAACGTGCAGACCTTCAAGTTCAAGATTCCCGCATCCACCCCCGCAGGCCAGTACCTCCTTCGCATTGAGCATATTGCGTTGCACGGCGCGTCCAGCACAGCTGGTGCTCAATTCTACATTAGCTGCGCCCAGATCAACGTCACCGGAGGCGGCTCAGGAAACCCGTCCAAGGTTTCTATCCCGGGCATCTACTCAGCGACCGACCCAGCAATCTTGATCAACATCTACTGGCCACCTGTCACCAGCTACACTCCCCCCGGCCCCGCGGTCTGGTCCGGCTGA

>RS60

ATGAATTTCAGCACAGCAACTATCCTCGCCGTCTTGGCTTTGACCCCTTTAGTTTTGGGTCTCCATGGTGTCAAAATCATGGCGAAAGATGGGCTCAATGCCCAAATCAAGGCCAAAGGCAAGATGTACTTTGGTACTTGTACCGATTATAGCTTGTTTACTAACCCCGCGAACGCTGCCATCATCCACTCCGACTTTGGTCAGCTCACGCCAGAGAACAGTGCGAAGTGGGATGCGACTGAGCCCTCGAGGGGAAAGTTCGAATTTGGGAGATTTGACACTTTTGTGAAATTCGCTGAAAACTATCACAAACTGATTCGTGGACACGCCTTTGTCTGGCACTCCCAGCTGGCACCCTGGGTAAACACCATCAGCGATTCCGGCACGCTCACCTCAGTCATCGAGCACCATATTACCACGATCGGAACCCGCTACAAGAGCAAGATCTACGCCTGGGACGTGGTTAACGAGATCTTCAACGAGGATGGCAGCCTGCGCTCTTCCGTGTTCAGCCGTGTTCTCGGCGAGAAATTTGTGGCTATTGCGTTCAAGGCTGCCCGCGCTGCCGATCCGTCAGCCAAGCTCTATATCAATGAGTACAACCTCGAATCTAACAGTCGCAAGCGCAACGCCCTCATTGATCTAGTCAAGCGTGAGAAGGAGGCTGGAACCCCCATCGATGGTATCGGCAGCCAAACACACCTCCTAGCCGGCCAGGCTGGTGGAGTTCAAGCCGCGCTGACTGAACTCGCGAACTCTGGGGTGCATGAAGTTGCGATTACCGAGCTCGATATTGCCGGTGCTTCGGGTAATGACTATGCTACGGTAGTCAAGGCATGCCTGGCCGTTTCCAAGTGCGTCGGAGTGACGGTCTGGGGGATCTCCGACAAGGACTCTTGGCGTTCCAGCACGAACCCTCTGTTGTTCGATGGCAACTACCATAAGAAGCCCGCTTATGACTCGGTCAGCAGTGCCCTGGCGCCAAGAAGGATATCTAGGCTTGCTAGGTCGCAAAGGGAATACTAA

>RS91

ATGAGGACCTTCGGAATTGTCATCGCTGTTGCTCTTTCCCTCTTCCAGGGCTCATTTGCGGCTCCCGCCATCAAGGCTAGGGATTGCCAAGTTATCCCTTCGACTGCGAACGCAGCTGTCCGTGATCAAGTCTACCGCATTACTCAGTCTCGCGCTGTCACCGCCAAGGTCTTACTCAGCACTTTCGAGACTGCATGGATCGAGTCCCATGTCAACAACTTGAATTGTGGCGACCAGGACTCCATTGGAGTCTTCCAACAGCGCCCTAGCCAAGGATGGGGATCCTACGACCAAATCATGAACGTCGACTACAGTACTAACAAGTTCCTCGACCAAGCGATTGTCAACGACAGGAACAACCCAGGATACACCGCCGGCCAACTCGCTCAGTCCGTTCAGCGCTCCGAGTTTCCTGATCGTTATGACCAAGCTCAAGGTACTGCCCAGGAGTTGATCAACCAGGCTCGTGCCTCTGTCGGAGGAATCACCCCTGGTAACGGCAAGTGCGCTGGTGTCGGCGCTTACGGTTCCGCCACGGTCTACACCGGAGGGCAAAAGTGTACCTATGGAGGACACTTGTGGACCGCGAAATGGTGGACTCAGTACGAGACTCCCAGCACTGGTGGCAGCGGTGTCTGGCAAGATGACGGTGCTTGCTAG

>RS95

ATGTTGTTTGCTTCTCTCGTCTCGTTTGTTGCGCTCGCAGCTGGTGTTAATGCCCACGGATATGTAGACAAGATCATTTGTGATGGCAAGACCTTCAACGGTCCCATCCCGGGCGAGACAAACCCCAAGTCTCCGATCCGCCGTATCAGCACCATTGATCCGTACAAGAGACCTGCTGGCTCTGGCATCACCTGCGGAGAGAACGCTAAAGCTGCCTCTATGGTCGCTCCCATCACCGCGGGCTCCGACTTGACCTTGTCGTGGGTCGCACACCCAAACCAGAAGTGGCCTCACGAGATGGGACCTCTCATCACTTACATGGCCAAGGTCCCCGCTGGGCAGACTGCCGACAAGTTTGATCCGTCCAAGGGTGATTTCTTCAAGGTTCACCAGGAAGGTCAGGAAGGCAAAAAGTGGTATCTCGAGCGTTTGATGAAACTCGGCACTACCATGTCTGTCCCGATCCCCAAGGAACTCGAAGACGGCCACTACATCATGCGCCACGAGATTATCGCTCTCCATCTCGCAGACCAGAAAGGCGGCGCCGAGTTTTACACCAGCTGCTTCCAGTTGAACGTCACTGGGGGCACCGGCACCGCCAAGACCACCGCGTCCGAGACCGTCCGTTTCCCGGGTGCGTACAGCGCCACCGACCCTGGCATTTACGTTCCCAAGGTCTTCGACTCGGGTTTCAAGTACAACTTCCCTGGCCCTGCGATTGCGACATTTGCCAATACCGGAAATTCGACCAAGCCTGACACACCTGCGACCGAGTCTTCTGTTCCGACCGCTGTTACTTCTTCTGCACCTGCTCCGACCTCGACTGGGGAGCCTGATGATGACGAGTGTGGTGCTGAGGAGCCTACTGCTACCGAGGATGACTGCACCGATGAGGACCCTGTTACCGAGTCCGAGTCTACTTCCTCGACGGCCAAGTACCACAAGCCGACTGGCAAGTCCCACAAAGACCGCCGGCACAACCGCTCTTGGACGTCGCGTCACCTTGGTGTTCGCAAGTAA
